# Supplementary material for: Characterization of GABA-Transaminase Gene from Mulberry (Morus multicaulis) and Its Role in Salt Stress Tolerance
Source: Genes (Basel). 2022 Mar 12;13(3):501. doi: 10.3390/genes13030501 (PMC8954524; doi:10.3390/genes13030501)
Supplement: Supplementary file 1 [file genes-13-00501-s001.zip › genes-1628862-supplementary.pdf]

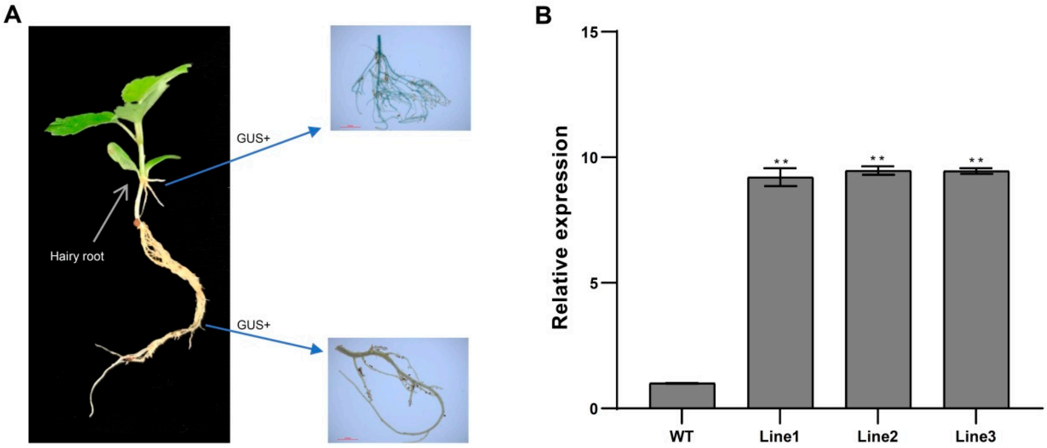

Figure S1. Identification of transgenic MuGABA-T mulberry hairy roots. (**A**) GUS staining identification of the hairy roots induced. (**B**) MuGABA-T abundance analysis by qRT-PCR. The relative gene expression was evaluated using comparative Ct method and the Actin gene was used as a reference gene. The value is the average of three biological replicates ± SD. WT: Hairy roots induced with K599 containing empty vector. L1-3 represents different transgenic MuGABA-T hairy root lines. **Asterisk indicates significant difference ($p < 0.05$).
